# TRPM7 kinase regulates α-cell proliferation and glucagon production in mice

**DOI:** 10.1016/j.molmet.2026.102317

**Published:** 2026-01-09

**Authors:** Severin Boulassel, Pascale C.F. Schreier, Andreas Beck, Hyeri Choi, Anna M. Melyshi, Peter S. Reinach, Megan Duraj, Mikhail Vinogradov, Bibiazhar Suleimen, Johanna Berger, Katharina Jacob, Andreas Breit, Susanna Zierler, Ingrid Boekhoff, Thomas Gudermann, Noushafarin Khajavi

**Affiliations:** 1 Walther Straub Institute of Pharmacology and Toxicology, Faculty of Medicine, LMU Munich, Munich, Germany; 2 Institute of Experimental and Clinical Pharmacology and Toxicology, Saarland University, Homburg, Germany; 3 Wenzhou Medical University, Ophthalmology Department, Wenzhou, PR China; 4 Institute of Pharmacology, Johannes Kepler University Linz, Linz, Austria; 5 German Center for Lung Research, Munich, Germany

**Keywords:** TRPM7, Glucagon secretion, α-cell identity, α-cell proliferation, mTOR signaling

## Abstract

**Objectives:**

Glucagon is essential for maintaining glucose homeostasis, yet the molecular mechanisms governing α-cell function remain incompletely understood. Transient receptor potential melastatin 7 (TRPM7) is a ubiquitously expressed ion channel with an intrinsic kinase domain, which regulates the mammalian target of rapamycin (mTOR) signaling in various cell types. Given the central role of mTOR in α-cell regulation, this study investigates how TRPM7 influences α-cell biology and examines whether its function is modulated through interaction with the mTOR signaling pathway.

**Methods:**

Islets were isolated from wild-type (WT) mice and mice lacking TRPM7 kinase activity (*Trpm7*^*R/R*^). Functional analyses included Bio-Plex assays, RNA sequencing, glucagon ELISA, qRT-PCR, Western blotting, immunocytochemistry, and patch-clamp recordings. αTC1c9 cells were used as a murine α-cell model. NS8593, a small synthetic compound, was used as a potent TRPM7 inhibitor.

**Results:**

*Ex vivo* analysis revealed impaired mTOR signaling in *Trpm7*^*R/R*^ islets. *Trpm7*^*R/R*^ islets secreted less glucagon in response to various secretagogues compared to WT controls. This reduction was partially caused by diminished glucagon content due to downregulation of key transcriptional regulators of glucagon biosynthesis, including *Gcg* and *Mafb*. Morphological analysis identified reduced proliferation and enhanced apoptosis of *Trpm7*^*R/R*^ α-cells. Similarly, pharmacological inhibition of TRPM7 impaired mTOR signaling, suppressed α -cell identity, and α-cell proliferation in both WT islets and αTC1c9 cells.

**Conclusions:**

Loss of TRPM7 kinase function impairs mTOR signaling, leading to reduced α-cell proliferation and glucagon secretion. Our findings show that the TRPM7 kinase/mTOR signaling pathway axis is a critical regulator of α-cell function in mice.

## Highlights

• TRPM7–mTOR coupling is indispensable for regulation of α-cell function and glucagon secretion.
• Loss of TRPM7 kinase suppresses *Gcg* and *Mafb* expression, impairing α-cell identity.
• Ablation of TRPM7 kinase reduces α-cell proliferation and enhances α-cell apoptosis.
• TRPM7–mTOR crosstalk emerges as a pivotal regulator of α-cell biology and glucose homeostasis.

## 1 Introduction

The rising incidence of obesity and diabetes represents a global health crisis, reaching epidemic levels in some populations and collectively accounting for over 1.4 million deaths annually [1]. While β-cell dysfunction has traditionally been the central focus of diabetes research, growing attention is now being directed towards delineating the involvement of pancreatic α-cells in regulating glucose metabolism in both health and disease [[2], [3], [4]]. Pancreatic α-cells are the primary source of glucagon, a hormone best known for its ability to raise blood glucose levels by counteracting the hypoglycemic effects of insulin. The inability of α-cell to secrete adequate glucagon in response to hypoglycemia is a significant barrier to achieving optimal glucose control in patients with diabetes [5,6]. Beyond this classical role, glucagon exerts a wide range of metabolic effects, particularly regulating amino acid metabolism, stimulating fatty acids oxidation and increasing energy expenditure [7,8]. In recent years, innovative approaches targeting glucagon receptor pathways through novel strategies, such as receptor antagonists, multi-agonists, and hepatic signaling modulators, offer a promising avenue for restoring metabolic balance. Thus, clarification of the molecular mechanisms regulating glucagon levels and α-cell function may provide deeper insight into the pathophysiology of abnormal glucose metabolism in diabetes and identify novel targets for improving therapeutic management of this disease.

Previous studies highlight the pivotal role of the mammalian target of rapamycin (mTOR) signaling pathway in regulating pancreatic α-cell function. Specifically, mTOR complex 1 (mTORC1) has emerged as a key regulator of α-cell mass and glucagon secretion, as evidenced by reduced α-cell proliferation in mice treated with the mTORC1 inhibitor rapamycin [9]. Moreover, studies using α-cell-specific knockout of Raptor, an essential scaffolding protein for mTORC1, demonstrated impaired glucagon release in response to various secretagogues [2]. Accumulating evidence suggests that the transient receptor potential melastatin 7 (TRPM7) channel regulates mTOR signaling across various cell types. TRPM7 is a ubiquitously expressed membrane protein composed of a divalent cation-selective channel fused to a protein kinase domain. The kinase activity of TRPM7 is essential for neutrophil recruitment and function, primarily through modulation of the Akt/mTOR signaling pathways [10]. In lymphocytes, TRPM7 deficiency leads to attenuated mTORC1 signaling, as indicated by decreased phosphorylation of the p70 ribosomal protein S6 kinase (S6K) [11]. Moreover, TRPM7 kinase domain activity regulates the differentiation of murine T lymphocytes into proinflammatory TH17 cells, a process that is also dependent on AKT/mTOR signaling pathways [12,13].

TRPM7 is highly expressed in both human and murine islet cells [14]. We recently identified TRPM7 kinase as a critical regulator of glucose metabolism in mice. Notably, mice lacking TRPM7 kinase exhibited pronounced glucose intolerance and hyperglycemia [15,16]. While this metabolic impairment was partially attributed to a diminished insulinotropic response in β-cells, the contribution of α-cells to this phenotype remained unclear. Given that TRPM7–mTORC1 signaling interactions exist in multiple cell types, and mTORC1 is a well-established regulator of α-cell function and glucagon secretion, the specific role of the TRPM7 channel and its kinase domain in this regulatory network warranted further investigation. To address this, we employed a genetically engineered mouse model harboring a single point mutation at the active site of the TRPM7 enzyme (*Trpm7*^*R/R*^). Our results provide compelling evidence that TRPM7 kinase plays a significant role in regulating α-cell proliferation and glucagon secretion by modulating mTOR signaling pathway activity. Notably, pharmacological inhibition of TRPM7 in both wild-type islets and αTC1c9 cells (a mouse α-cell line) impaired α-cell function and recapitulated the defects observed in *Trpm7*^*R/R*^ mice, further supporting the critical role of TRPM7 in maintaining α-cell homeostasis.

## 2 Methods

### 2.1 Mouse strains and genotyping procedures

*Trpm7tm1.1Mkma* C56BL/6 (K1646R, *Trpm7*^*R/R*^) mice were provided by Masayuki Matsushita (Okayama University Medical School, Okayama, Japan). Mice were backcrossed to C57BL/6 (≥6 generations). Mice were maintained under standard conditions in individually ventilated cages at the animal facility of the Walther Straub Institute of Pharmacology and Toxicology, LMU Munich, Germany. To generate age- and sex-matched experimental cohorts, heterozygous K1646R mice were interbred to obtain homozygous WT and homozygous *Trpm7*^*R/R*^ littermates. Genomic DNA for genotyping was isolated from ear biopsies using the Mouse Direct PCR Kit (Biotool). PCR amplification was carried out with allele-specific primers obtained from Metabion. Genotyping procedures for *Trpm7*^*R/R*^ mice were performed according to previously published protocols [17]. Sequence information is reported before [15]. For investigation of blood parameters, blood was collected after euthanasia using lithium heparin micro sample tubes (Sarstedt), immediately cooled on ice, centrifuged at 2,000×*g* and 4 °C for 10 min, and plasma stored at −80 °C. All animal experiments were performed in accordance with the EU Animal Welfare Act and were approved by the District Government of Upper Bavaria, Munich, Germany, on animal care (permit no. 55.2–2532. Vet_02-19-035).

### 2.2 Islet isolation and glucagon secretion assay

Pancreatic islets were isolated from male and female mice aged 8–30 weeks, as previously described [18]. Briefly, the pancreas was perfused via the common bile duct with 3 mM collagenase P (0.5 mg/mL; Roche) in HBSS supplemented with 25 mM HEPES and 0.5% (w/v) BSA. Isolated islets were recovered for 48 h in RPMI 1640 medium (Thermo Fisher Scientific) at 37 °C in a humidified atmosphere containing 5% CO_2_. Following recovery, islets were used for functional assays. Prior to glucagon secretion measurements, islets were pre-equilibrated for 1 h in Krebs–Ringer bicarbonate (KRB) buffer (115 mM NaCl, 4.5 mM KCl, 1.2 mM KH_2_PO_4_, 2.6 mM CaCl_2_, 1.2 mM MgSO_4_, 10 mM HEPES, 20 mM NaHCO_3_, 0.1% BSA, pH 7.4) containing 2.8 mM glucose. Glucagon secretion was assessed in 12-well plates with 600 μL KRB per well (8 islets/well), using five independent experiments performed in triplicate. After a 1 h preincubation in KRB with 2.8 mM glucose, islets were stimulated for 1 h with either 2.8 mM glucose, 20 mM glucose, 20 mM arginine or 30 mM KCl. Glucagon released into the supernatant was quantified using a glucagon ELISA kit (Mercodia, Uppsala, Sweden). To determine total glucagon content, groups of 10 islets were lysed in RIPA buffer, and glucagon levels were measured using the same ELISA kit. Plasma glucagon was also quantified by a glucagon ELISA kit (Mercodia, Uppsala, Sweden).

### 2.3 Bio-Plex pro cell signaling assay

Murine pancreatic islets were washed and lysed in MILLIPLEX® MAP Lysis Buffer (Merck). Total protein concentration was determined using the Pierce™ BCA Protein Assay Kit (Thermo Fisher Scientific, catalog no. 23225), and lysates were stored at −80 °C until further analysis. Samples were processed and analyzed according to the manufacturer's instructions for the 2plx Phospho/Total mTOR MAG Kit (Merck, catalog no. 48-625MAG) and the MILLIPLEX® MAP β-Tubulin Total Magnetic Bead MAPmate (Merck, catalog no. 46-713MAG).

### 2.4 Western blot analysis

Western blotting was performed as previously described. Briefly, 20 μg of total protein per sample was separated on 12.5% Tris–HCl SDS-PAGE gels and transferred onto PVDF membranes (Millipore). Membranes were blocked for 1 h at room temperature with 5% BSA or nonfat dry milk in Tris-buffered saline containing 0.1% Tween-20 (TBS-T), followed by overnight incubation at 4 °C with primary antibodies (listed in Supplemental Table 1). After washing, membranes were incubated with HRP-conjugated secondary antibodies (Supplemental Table 1) for 1 h at room temperature. Signal detection was performed using chemiluminescent peroxidase substrate (Sigma Alderich), and chemiluminescence was visualized with a ChemiDoc MP Imaging System (Bio-Rad). Histone H3 was used as a loading control.

### 2.5 RNA isolation and cDNA synthesis

Total RNA was isolated from pancreatic islets and αTC1c9 cells using the RNeasy Mini Kit (QIAGEN) according to the manufacturer's instructions. Complementary DNA (cDNA) was synthesized from the extracted RNA using the QuantiTect Reverse Transcription Kit (QIAGEN), following the manufacturer's protocol.

### 2.6 Quantitative real-time PCR (qRT-PCR)

qRT-PCR was performed in triplicate using the LightCycler® 480 II (Roche). Amplification was carried out over 40 cycles with the following thermal profile: 95 °C for 10 s and 54 °C for 20 s. Primers were designed using Primer3 and validated for linear amplification using serial dilutions of cDNA prior to analysis of experimental samples. Primer sequences are listed in Supplemental Table 2.

### 2.7 RNA-Seq studies

RNA-Seq data have been deposited in the NCBI Gene Expression Omnibus (GEO) under accession number GSE218030. Total RNA was extracted from isolated pancreatic islets of *Trpm7*^*R/R*^ mice and their control littermates, all of which had been maintained on a high-fat diet for 16 weeks. Bulk RNA sequencing of freshly isolated murine islets was performed using the NEBNext® Single Cell/Low Input RNA kit (Illumina®) for library preparation, optimized for low-input RNA, followed by sequencing on the NovaSeq® 6000 platform with 2 × 100 bp paired-end reads. Library preparation, amplification, and sequencing were conducted using exclusion amplification (ExAmp) chemistry and NovaSeq Control Software v1.6.0. Differential expression analysis was performed using pairwise comparisons, with P values calculated via the Wald test. To control for false-positive results, both false discovery rate (FDR)-adjusted and Bonferroni-corrected P values were computed. Genes or transcripts were considered significantly differentially expressed if the FDR-adjusted P value was ≤0.05 and the fold change was ≥2.

### 2.8 Immunostaining of intact islets

Freshly isolated intact islets were fixed in 4% paraformaldehyde for 10 min at room temperature and permeabilized with 0.5% Triton X-100 in PBS. After blocking with 4% BSA supplemented with goat serum for 1 h, islets were incubated with primary antibodies diluted in 2% BSA/PBS for 2 h at room temperature. Following extensive washes, fluorophore-conjugated secondary antibodies were applied for 1 h in the dark. Nuclei were counterstained with DAPI, and islets were mounted in antifade medium. Confocal images were acquired at the equatorial plane of intact islets to ensure consistency in quantification.

### 2.9 Morphological analysis

Pancreata were collected and fixed in 4% paraformaldehyde in PBS for 22 h at 4 °C. Tissues were then cryoprotected in 30% sucrose in PBS at 4 °C until fully equilibrated, embedded in optimal cutting temperature (OCT) compound, and frozen for cryosectioning. Immunofluorescence staining on 10 μm cryosections of pancreas was performed to assess pancreatic islet morphology. Antibodies and their working dilutions are listed in Supplemental Table 2. Digital imaging fluorescence microscopy of the pancreas was performed using a scanning platform (MetaSystems) with an Imager Z.2 microscope (Carl Zeiss MicroImaging, Inc.). Quantitative image analysis of islet morphology was performed using ImageJ (NIH). For quantification of the percentage of glucagon-positive cells per islet area, all images were analyzed by an investigator linded to the experimental groups. Individual islets were outlined manually based on the insulin and glucagon staining to define the islet area. The glucagon-positive area within each islet was determined, and the ratio of glucagon-positive area to total islet area was calculated using ImageJ. At least 5 islets per pancreas were analyzed, and the mean value per animal was used for statistical analysis.

### 2.10 Measurement of α-cell proliferation and apoptosis

To assess α-cell proliferation, pancreatic islets were co-immunostained for detection of glucagon and Ki67, the proliferation marker. The number of Ki67^+^ glucagon^+^ cells was quantified and expressed as a percentage of the total number of glucagon^+^ cells per islet. Antibodies and their working dilutions are listed in Supplemental Table 2. For the analysis of α-cell apoptosis, the ApopTag Red *In Situ* Apoptosis Detection Kit (Merck) was used according to the manufacturer's instructions. TdT-mediated dUTP nick-end labeling (TUNEL)^+^ glucagon^+^ cells were counted and normalized to the total number of glucagon^+^ cells within the islet. To assess cell proliferation and apoptosis in WT islets and αTC1c9 cells following pharmacological inhibition of TRPM7, samples were preincubated with 30 μM NS8593 for 48 h. Samples were co-stained then for glucagon and Ki67 or ApopTag Red *In Situ* Apoptosis Detection Kit. Ki67^+^ glucagon^+^ cells or TUNEL^+^ glucagon^+^ cells were counted and normalized to the total number of glucagon^+^ cells.

### 2.11 Cell culture

αTC1c9 cells (α-cells) were purchased from Hölzel. Cells were cultured at 37 °C with 5% CO_2_ in DMEM (Gibco) supplemented with 10% FBS (Gibco), penicillin (100 U/mL) and streptomycin (100 μg/mL) (Gibco), 15 mM HEPES (Gibco), 0.1 mM non-essential amino acids (Gibco), and 0.02% BSA (Roth). For patch clamp experiments α-cells were plated on glass cover slips.

### 2.12 Electrophysiological recordings

Whole-cell membrane currents were recorded using an EPC-9 amplifier (HEKA Electronics). Patch pipettes were pulled from glass capillaries GB150T-8P (Science Products) at a vertical Puller (PC-10, Narishige) and had resistances of 3–4 MΩ when filled with internal solution which comprised of (in mM) 120 Cs-glutamate, 8 NaCl, 10 HEPES, 5 Cs-EDTA, 10 Cs-EGTA, pH 7.2. The external solution contained (in mM) 140 NaCl, 2 MgCl_2_, 1 CaCl_2_, 10 HEPES, 10 glucose, pH 7.2. Divalent-free solution (DVF; CaCl_2_ and MgCl_2_ were omitted from external solution and 5 mM Na-EDTA was added) or external solution with 30 μM NS8593 were directly applied onto the patch-clamped cell via an air pressure-driven (MPCU, Lorenz Meßgerätebau) application pipette. All solutions had an osmolality of 290–310 mOsm. Every 2 s voltage ramps of 50 ms duration spanning from −100 mV to 100 mV were applied from a holding potential (V_h_) of 0 mV using the PatchMaster software (HEKA). All voltages were corrected for a liquid junction potential of 10 mV and currents were filtered at 2.9 kHz and digitized at 100 μs intervals. Before each voltage ramp, capacitive currents and series resistance were determined and corrected by the EPC9 automatic capacitance compensation. Inward and outward currents at −80 and + 80 mV were extracted from each individual ramp current recording and amplitudes were plotted versus time. Current–voltage (IV) relationships were extracted at indicated time points. To obtain the net developing current, basic currents were subtracted from single IVs. All currents were normalized to the initial size i.e. capacitance of the cell to obtain current densities (pA/pF).

### 2.13 Statistics

Data are presented as mean ± SEM. A P value less than 0.05 was considered statistically significant. Graphical representations, curve fittings, and statistical analyses were performed using IGOR Pro (version 6.31) and GraphPad Prism software (version 9.0.1). For comparisons between two groups, unpaired two-tailed Student's *t* test was used for normally distributed data, while the Mann–Whitney test was applied for nonparametric distributions. When comparing three or more groups, one-way ANOVA followed by Bonferroni's multiple comparisons test was used for parametric data.

## 3 Results

### 3.1 Genetic loss of TRPM7 kinase impairs mTOR signaling in murine islets

**Figure 1:**
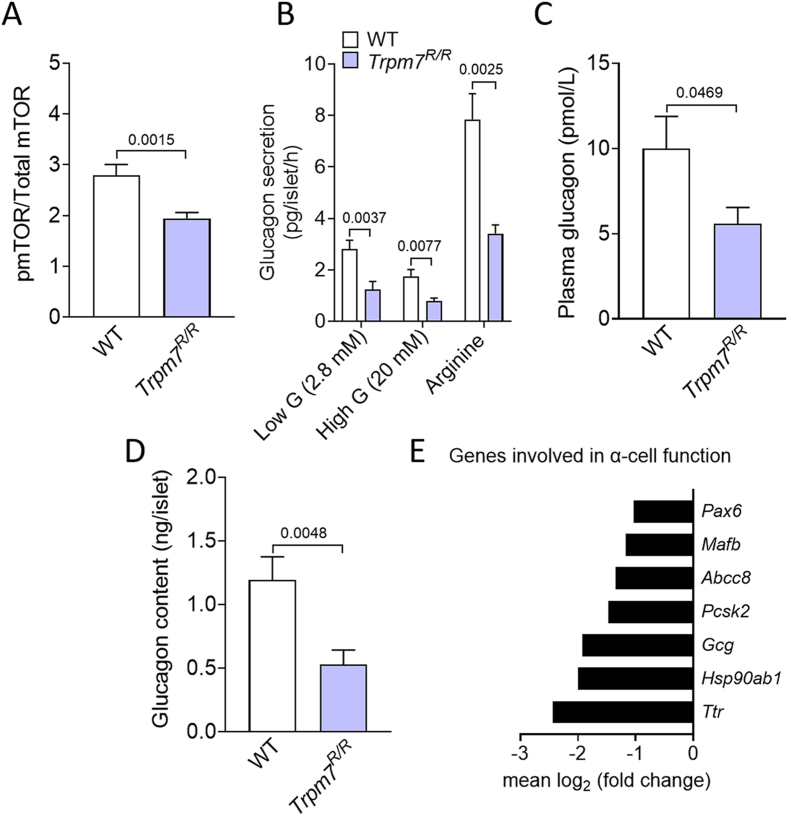
**TRPM7 kinase disruption impairs mTOR signaling and glucagon secretion in murine islets. (A)** Assessment of the activity of the cell signaling molecules mTOR using Bio-Plex assay and phospho-specific antibodies on lysates of isolated islets from WT and *Trpm7*^*R/R*^ mice (n = 8, measured in duplicates, 8 mice per genotype). **(B)** Glucagon secretion (pg/islet/h) in isolated islets of male and female *Trpm7*^*R/R*^ and control littermate mice. Islets were incubated for 1 h in the presence of low glucose (2.8 mM), high glucose (20 mM) or 20 mM Arginine (n ≥ 3 mice per genotype, measured in duplicate). **(C)** Plasma levels of glucagon (pmol/L) (n ≥ 8 mice per genotype) in freely fed male and female *Trpm7*^*R/R*^ mice and control littermates. **(D)** Total glucagon content (ng/islet) of WT versus *Trpm7*^*R/R*^ islets. At least 15 groups of 10 size-matched WT and *Trpm7*^*R/R*^ islets were compared. **(E)** Summary of the genes that were downregulated in *Trpm7*^*R/R*^ mice. For RNA-Seq analysis, islet RNA was collected from *Trpm7*^*R/R*^ mice and the control littermates (n = 3 mice per genotype, age: ∼24 weeks). Differentially expressed genes (DEGs) were identified (P < 0.05) by using EdgeR method. DEGs are expressed as log_2_ fold change over control with an adjusted P value for each gene. Data show means + SEM, and statistical differences were assessed by unpaired 2-tailed Student's t test. P values are shown above the bars.

To explore the potential involvement of TRPM7 kinase in regulating the mTOR signaling pathway in pancreatic islets, we employed a bead-based Bio-Plex assay to quantitatively assess the phosphorylation status of key mTOR signaling proteins in islets isolated from 24-week-old *Trpm7*^*R/R*^ mice. *Trpm7*^*R/R*^ islets exhibited a significant reduction in mTOR signaling activity, with phosphorylation of mTOR decreased by up to 30% compared to WT controls (Figure 1A).

### 3.2 Inactivation of TRPM7 kinase reduces glucagon secretion and content in murine islets

Since mTOR signaling is a key regulator of α-cell function, we next examined whether its impairment in *Trpm7*^*R/R*^ mice disrupts the α-cell function. To this end, we assessed the response of isolated islets to various secretagogues. Glucagon secretion in response to 2.8 mM glucose and 20 mM glucose and 30 mM arginine was reduced by up to 50% in *Trpm7*^*R/R*^ islets compared to WT controls (Figure 1B). To determine whether this *ex vivo* defect is reflected *in vivo*, we measured plasma glucagon levels in both genotypes. *Trpm7*^*R/R*^ mice exhibited significantly lower plasma glucagon levels compared to their WT littermates (Figure 1C). We further quantified the glucagon content in islets isolated from both genotypes. We observed a significant reduction in total glucagon content in *Trpm7*^*R/R*^ islets compared to their control littermates (Figure 1D). To assess whether the diminished glucagon secretion is attributable to impaired Ca^2+^ signaling or to a defect in the exocytosis, we depolarized islets with high extracellular potassium. Strikingly, glucagon secretion elicited by 30 mM KCl was comparable between WT and *Trpm7*^*R/R*^ islets when normalized to glucagon content, indicating that the exocytotic machinery downstream of membrane depolarization is preserved (Supplemental Fig. 1).

### 3.3 TRPM7 kinase disruption reduces expression levels of genes involved in α-cell function

Transcriptomic profiling of islets isolated from *Trpm7*^*R/R*^ and WT mice after 16 weeks on a high-fat diet revealed marked alterations in key genes governing α-cell function. Notably, *Gcg* expression was significantly downregulated in *Trpm7*^*R/R*^ mice. In line with this decline, *Pcsk2*, the enzyme responsible for processing preproglucagon to mature glucagon [19], also showed reduced expression. Additionally, *Pax6*, a transcription factor essential for *Gcg* expression and α-cell differentiation, was downregulated. Notably, *Mafb* and *Ttr*, two well-established α-cells markers [19], exhibited decreased expression compared to WT controls. Furthermore, we also detected reduced expression of *Abcc8*, which plays a role in regulating membrane potential difference and glucagon secretion. Lastly, the expression of *Hsp90ab1*, a gene associated with endoplasmic reticulum stress responses diminished in *Trpm7*^*R/R*^ mice, suggesting possible involvement of cellular stress pathways (Figure 1E and Supplemental Table 3). Importantly, the expression level of K_ATP_ channel components (*Sur1* and *Kir6.2*) as well as autophagy-associated genes (*Ulk1* and *Ulk2*) remained comparable between genotypes, ruling out major contributions from these pathways to the α-cell dysfunction observed in *Trpm7*^*R/R*^ mice.

### 3.4 TRPM7 kinase domain is a critical determinant of α-cell proliferation and survival

**Figure 2:**
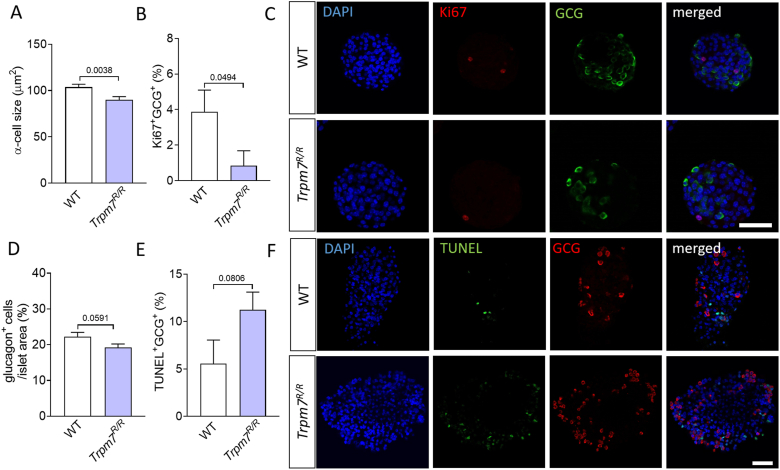
**TRPM7 kinase disruption impairs α-cell mass in mice. (A)** α-Cell size (≥10 islets, at least 3 mice per genotype) and **(B)** percentage of Ki67-positive cells from the population (100%) of the glucagon-positive cells per pancreatic islet in WT and *Trpm7*^*R/R*^ mice (n ≥ 30, 5 mice per genotype). **(C)** Confocal images of islets stained for DAPI (blue), glucagon (green), and Ki67 (red). **(D)** Percentage of glucagon positive cells per islet in WT and *Trpm7*^*R/R*^ mice (n ≥ 30, 5 mice per genotype). **(E)** Percentage of TUNEL-positive cells from the population (100%) of the glucagon-positive cells per pancreatic islet in WT and *Trpm7*^*R/R*^ mice (n ≥ 20, 5 mice per genotype). **(F)** Confocal images of TUNEL staining of isolated islets. TUNEL staining is shown in green, glucagon staining is shown in red, and nuclei (DAPI) are shown in blue. The scale bars represent 100 μm. Data in A, B, D and E show means + SEM, and statistical differences were assessed by unpaired 2-tailed Student's t test. P values are shown above the bars.

Given the well-established role of mTOR in regulating α-cell mass, we investigated the impact of TRPM7 kinase disruption on α-cell proliferation and survival. To this end, we performed morphological analyses of *Trpm7*^*R/R*^ and WT islets from mice aged over 30 weeks. *Trpm7*^*R/R*^ islets exhibited a significant reduction in α-cell size compared to control littermates (Figure 2A). Ki67 was used as a marker of proliferation. The proportion of Ki67^+^ α-cells was markedly lower in *Trpm7*^*R/R*^ islets, indicating impaired proliferative capacity (Figure 2B,C). Quantification of glucagon-positive area relative to total islet area indicated a decreased α-cell mass in *Trpm7*^*R/R*^ mice compared with WT controls, although this difference did not reach statistical significance (Figure 2D). TUNEL staining was employed to assess apoptosis and revealed an increase in apoptotic α-cells in *Trpm7*^*R/R*^ islets relative to controls (Figure 2E,F), suggesting that loss of TRPM7 kinase activity promotes α-cell apoptosis. Together, these findings indicate that TRPM7 kinase function is critical for maintaining α-cell growth and survival.

### 3.5 Pharmacological inhibition of TRPM7 attenuates rpS6 phosphorylation and lowers glucagon content in murine islets

**Figure 3:**
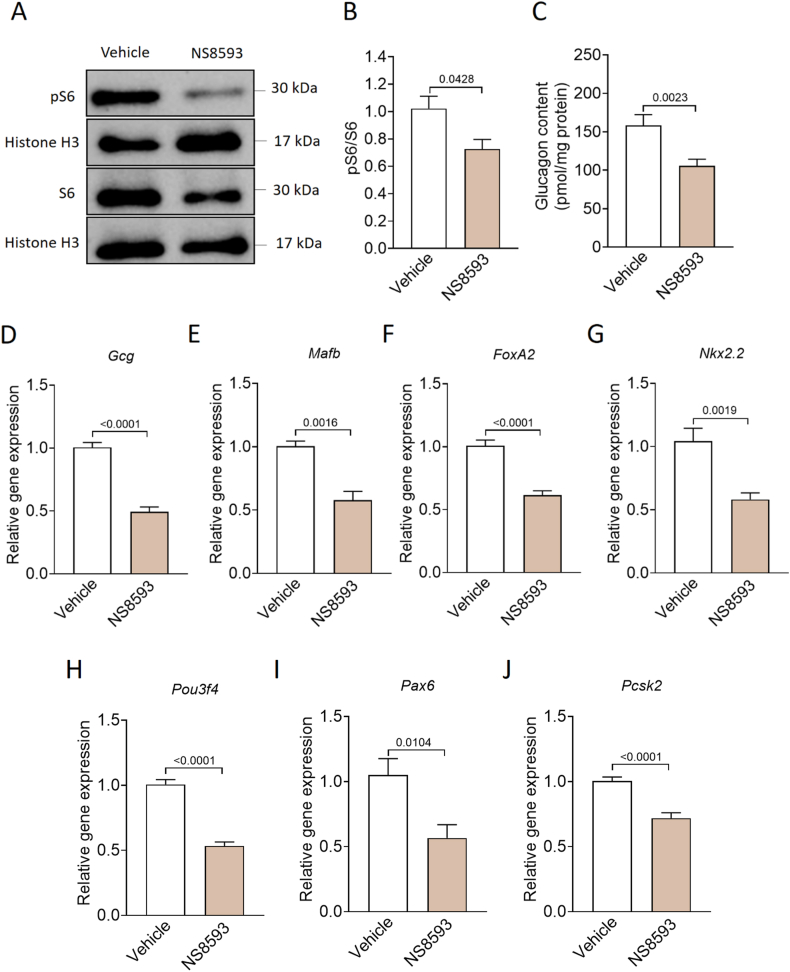
**Pharmacological inhibition of TRPM7 impairs rpS6 phosphorylation and reduces expression levels of genes involved in mediating α-cell identity. (A, B)** Western blot detection of the pS6 and S6 in lysates of purified WT islets treated with DMSO (Vehicle) or 30 μM NS8593 for 48 h (n ≥ 5 mice). Histone H3 was used as a loading control. **(C)** Total glucagon content (pmol/mg protein) of purified WT islets treated with DMSO (Vehicle) or 30 μM NS8593 for 48 h. At least 34 groups of 10 size-matched islets were compared. **(D**–**J)** Expression levels of *Gcg*, *Mafb*, *FoxA2*, *Nkx2.2*, *Pou3f4*, *Pax6*, and *Pcsk2* were analyzed by qRT-PCR using RNA isolated from WT islets treated with DMSO (Vehicle) or 30 μM NS8593 for 48 h (n ≥ 5 mice). Data show means + SEM, and statistical differences were assessed by unpaired 2-tailed Student's t test. P values are shown above the bars.

Multiple studies showed that treatment with the small molecule NS8593 recapitulates key cellular phenotypes observed following genetic inactivation of TRPM7 [20]. Building on our observations of reduced mTOR signaling and glucagon content in *Trpm7*^*R/R*^ islets, we next investigated whether pharmacological targeting of TRPM7 with NS8593 reproduces these effects on the phosphorylation status of ribosomal protein S6 (rpS6), a downstream target of mTOR, in WT islets. Western blot analysis revealed that WT islets exposed to 30 μM NS8593 for 48 h exhibit a 35% reduction in rpS6 phosphorylation at Ser240 compared to untreated controls (Figure 3A,B). We next quantified the glucagon content in WT islets exposed to 30 μM NS8593 and identified a marked reduction in total glucagon compared with vehicle-treated controls (Figure 3C).

### 3.6 NS8593 treatment alters transcriptional profile of α-cells and impairs α-cell proliferation

**Figure 4:**
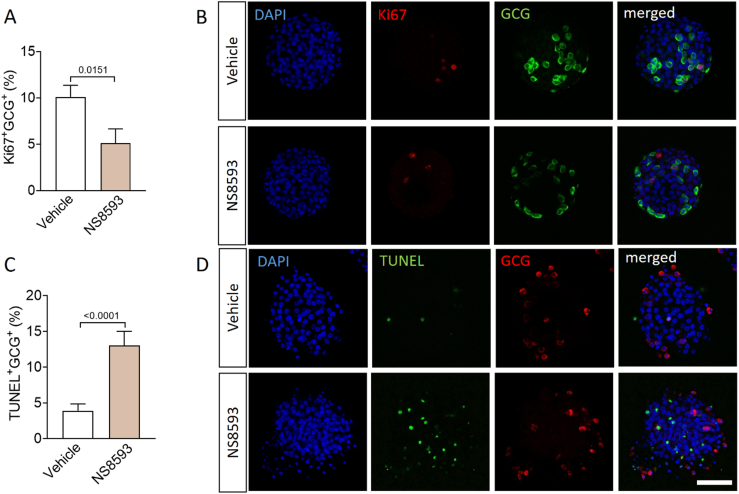
**TRPM7 inhibition impairs α-cell proliferation and enhances α-cell apoptosis in mice. (A)** Percentage of Ki67-positive cells from the population (100%) of the glucagon-positive cells per pancreatic islet in WT islets treated with DMSO (Vehicle) or 30 μM NS8593 for 48 h (n ≥ 30, 5 mice). **(B)** Confocal images of islets stained for DAPI (blue), glucagon (green), and Ki67 (red). **(C)** Percentage of TUNEL-positive cells from the population (100%) of the glucagon-positive cells per pancreatic islet in WT islets treated with DMSO (Vehicle) or 30 μM NS8593 (n ≥ 24, 5 mice) for 48 h. **(D)** Confocal images of TUNEL staining of isolated islets. TUNEL staining is shown in green, glucagon staining is shown in red, and nuclei (DAPI) are shown in blue. The scale bar represents 100 μm. Data in A and C show means + SEM, and statistical differences were assessed by unpaired 2-tailed Student's t test. P values are shown above the bars.

To assess the impact of pharmacological TRPM7 inhibition on the transcriptional profile of α-cells, we performed qRT-PCR analyses on WT islets following 48 h of exposure to 30 μM NS8593. Treatment with NS8593 resulted in a significant reduction in *Gcg* expression, a key gene involved in glucagon biosynthesis (Figure 3D). Moreover, the expression levels of *Mafb*, *FoxA2, Nkx2.2*, *Pou3f4*, *Pax6*, and *Pcsk2*, key transcription factors essential for α-cell development and maintenance, were markedly reduced in WT islets treated with NS8593 (Figure 3E–J). In addition, NS8593 treatment in WT islets led to a significant reduction in the proportion of Ki67^+^ α-cells, accompanied by a notable increase in TUNEL^+^ α-cells (Figure 4A–D).

### 3.7 Pharmacological inhibition of TRPM7 suppresses α-cell identity and reduces α-cell proliferation in αTC1c9 cells

**Figure 5:**
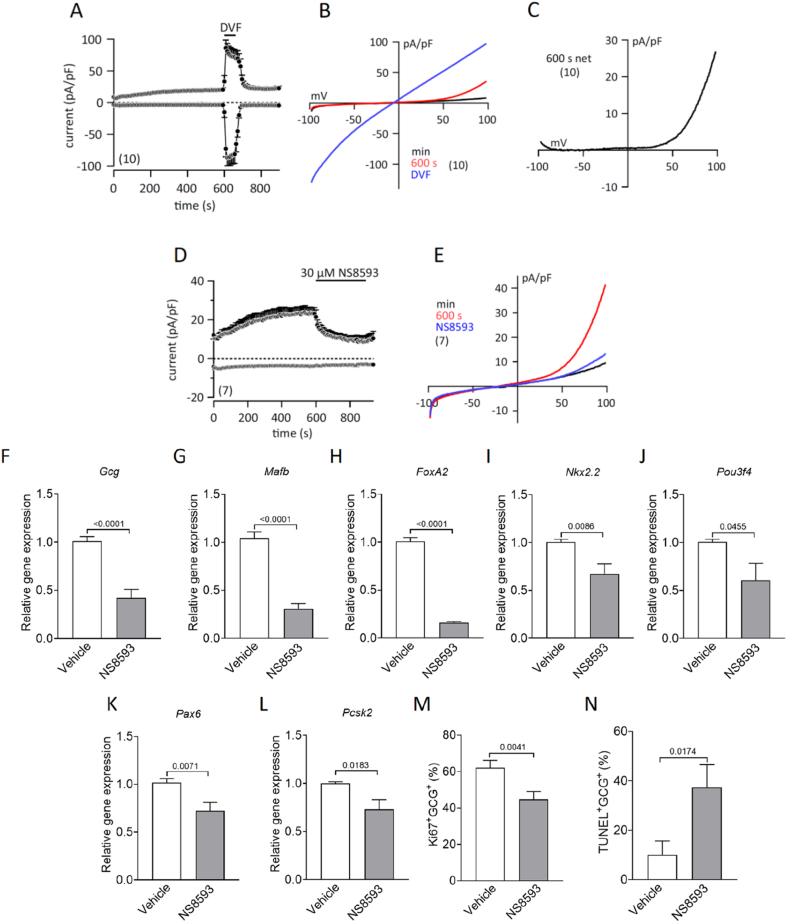
**Effects of pharmacological inhibition of TRPM7 onmembrane currents, gene expression, proliferationand apoptosis in αTC1c9 Cells. (A, D)** In- and outward currents at −80 mV and 80 mV, measured from αTC1c9 cells in the absence of intracellular Mg^2+^ plotted versus time. Divalent-free solution (DVF, A) or 30 μM NS8593 (D) were applied as indicated by the bars. Current-voltage relationships (IVs) of the minimal current (min, black), the current right before (600 s, red) and in DVF solution (DVF, blue), as well as the net current at 600 s (600 s net = 600 s minus min) from A are shown in **B** and **C**. IVs of the minimal current (min, black), the current right before (600 s, red) and at the end of NS8593 application (NS8593, blue) are shown in **E**. Data in A and D represent means ± S.E.M., IVs are means. Numbers of experiments are shown in parentheses. **(F**–**L)** Expression levels of *Gcg*, *Mafb*, *FoxA2*, *Nkx2.2*, *Pou3f4*, *Pax6*, and *Pcsk2* were analyzed by qRT-PCR using RNA isolated from αTC1c9 cells treated with DMSO (Vehicle) or 30 μM NS8593 for 48 h (n ≥ 3 independent experiments in triplicate). **(M)** Percentage of Ki67-positive cells from the population (100%) of the αTC1c9 cells treated with DMSO (Vehicle) or 30 μM NS8593 for 48 h (3 independent experiments, measured in duplicate). **(N)** Percentage of TUNEL-positive cells from the population (100%) of the αTC1c9 cells treated with DMSO (Vehicle) or 30 μM NS8593 for 48 h (3 independent experiments, measured in duplicate). Data show means + SEM, and statistical differences were assessed by unpaired 2-tailed Student's t test. P values are shown above the bars.

To eliminate potential paracrine influences or cell–cell interactions inherent to intact islets, we assessed pharmacological TRPM7 modulation in the αTC1c9 cell line. Functional TRPM7 expression was verified by patch-clamp recordings, which revealed whole-cell currents developing within 600 s in the absence of intracellular Mg^2+^. Removal of external divalent cations (DVF) markedly increased inward and outward currents in the current–voltage (I–V) relationship, consistent with TRPM7 channel properties (Figure 5A–C). In addition, the TRPM7-like whole-cell current, mediated upon depletion of intracellular Mg^2+^, was blocked by the TRPM7 inhibitor NS8593 (30 μM; Figure 5D,E). Treatment of αTC1c9 cells with NS8593 (30 μM) for 48 h led to a marked reduction in *Gcg* expression, as well as in the transcript levels of key α-cell identity genes, including *Mafb*, *FoxA2, Nkx2.2*, *Pou3f4*, *Pax6*, and *Pcsk2* (Figure 5F–L). Furthermore, NS8593 treatment resulted in a significant decrease in the proportion of Ki67^+^ αTC1c9 cells, alongside a marked increase in TUNEL^+^ αTC1c9 cells (Figure 5M, N, Supplemental Figs. 2 and 3). These findings indicate that the impaired transcriptional profile and reduced α-cell mass observed in pancreatic islets following TRPM7 disruption can be recapitulated in αTC1c9 cell line, thereby confirming a cell-autonomous role for TRPM7 in α-cell biology.

## 4 Discussion

In 1975, Roger H. Unger proposed the ground breaking hypothesis that an imbalance between glucagon and insulin plays a central role in the pathogenesis of diabetes mellitus [21]. In individuals with diabetes, glucagon secretion is often dysregulated in two major ways: a chronic elevation in glucagon levels, or relative hyperglucagonemia, which exacerbates hyperglycemia; and a diminished glucagon response to hypoglycemia, which impairs the body's ability to restore normal blood glucose levels. These abnormalities highlight the central role of α-cell dysfunction in the pathophysiology of diabetes [3,21,22]. Emerging therapeutic strategies, including glucagon receptor antagonists [23,24], dual GLP-1/glucagon receptor agonist [25], GLP-1/GIP/glucagon receptor triagonist [26,27] and modulators of hepatic glucagon signaling [28], show promise for correcting metabolic imbalances through targeted modulation of glucagon receptors. Thus, a deeper understanding of α-cell programming and function is crucial for optimally leveraging their therapeutic potential and advancing the development of more effective diabetes therapies.

We identify here a previously unrecognized role of TRPM7 kinase in maintaining postnatal α-cell function and regulating glucagon secretion. Consistent with earlier reports that TRPM7 overexpression induces hyperphosphorylation of ribosomal protein S6 (rpS6), a downstream effector of the PI3K/mTOR pathway [29], TRPM7 kinase inactivation in *Trpm7*^*R/R*^ islets reduced mTOR signaling, a pathway central to glucagon regulation during fasting, hypoglycemia, and glucoprivic stress [2,30]. Functionally, loss of TRPM7 kinase led to lower plasma glucagon levels, diminished glucagon secretion, and reduced islet glucagon content. Importantly, arginine-stimulated glucagon secretion was markedly attenuated in the absence of functional TRPM7 kinase. This defect is of particular significance given that nutrient availability, especially amino acids such as arginine, alanine, and glutamine, potentiates glucagon release through nutrient-sensing pathways, including mTORC1 [2,31].

Beyond its role in glucagon regulation, maintenance of α-cell mass critically depends on intact mTORC1 signaling [2,9,32]. Phosphorylation of rpS6 is indispensable for cell growth and proliferation [33,34]. Our morphological analysis revealed a significant reduction in α-cell size in *Trpm7*^*R/R*^ islets. In addition, genetic loss of TRPM7 kinase activity markedly decreased α-cell proliferation. Immunofluorescence analysis further demonstrated increased α-cell apoptosis, evidenced by a higher frequency of TUNEL-positive cells. This finding is consistent with previous observation in αRaptor^KO^ mice, where loss of mTORC1 activity induces progressive α-cell mass reduction through enhanced apoptosis and diminished proliferation. In αRaptor^KO^ mice, these defects emerge as early as one month of age and intensify over time, underscoring the sustained requirement for mTORC1 signaling in α-cell survival and maintenance [2]. While the reduction in glucagon content in *Trpm7*^*R/R*^ mice is likely attributable in part to diminution of α-cell mass, transcriptomic analysis further implicates transcriptional mechanisms. Specifically, we observed downregulation of the *Gcg* gene, along with key transcription factors involved in α-cell development and maintenance, including *Mafb*, *Ttr*, *Pcsk2*, and *Pax6.* Collectively, these findings indicate that the reduced glucagon content in *Trpm7*^*R/R*^ islets reflects the convergent effects of impaired glucagon biosynthetic capacity and loss of α-cell mass. Our data position TRPM7 kinase upstream of mTOR as a critical regulator of the α-cell phenotype, coordinating proliferative and anti-apoptotic pathway activity at levels that preserve α-cell function.

Although *in vivo* glucagon measurements were not performed in the present study, our *ex vivo* findings align with previous evidence demonstrating that disruption of the mTOR signaling pathway impairs α-cell function, thereby perturbing systemic glucagon regulation. Notably, both αRaptor^HET^ and αRaptor^KO^ mice exhibit markedly reduced glucagon secretion during insulin-induced hypoglycemia or glucoprivic challenges induced by 2-deoxy-d-glucose injection [2]. Similarly, rapamycin-treated mice display significantly diminished serum glucagon levels during insulin-induced hypoglycemia [30]. Together, these findings provide a mechanistic framework suggesting that perturbations in mTOR signaling pathway contribute to defective glucagon secretion and the consequent dysregulation of glucose metabolism *in vivo*.

We recently described a reduced β-cell proliferation in *Trpm7*^*R/R*^ mice [15,16]. Nevertheless, we believe this effect is independent of the impaired α-cell mass. A previous study reported no changes in β-cell mass in αRaptor^KO^ mice, suggesting that mTOR-linked α-cell loss has no significant impact on β-cell phenotypic maintenance under normal conditions [2]. In addition, there is evidence that mTORC1 inhibition, either via amino acid deprivation or rapamycin treatment, promotes crinophagic degradation of glucagon-containing granules. This degradation leads to reduced intracellular and secreted glucagon levels, even under hypoglycemic conditions [30]. While our transcriptomic analysis showed comparable expression of canonical autophagy genes (*Ulk1* and *Ulk2*) in WT and *Trpm7*^*R/R*^ islets, crinophagic degradation can occur independently of ULK-mediated macroautophagy. Therefore, we cannot exclude a potential contribution of crinophagy to the reduced glucagon secretion observed in *Trpm7*^*R/R*^ islets, and future studies using targeted genetic models will be required to define its precise role in TRPM7-dependent regulation of α-cell function.

TRPM7 has emerged as a novel therapeutic target, as its dysfunction has been associated with various cellular impairments and diseases. In recent years, several laboratories have identified small molecules capable of modulating either the channel or kinase activity of TRPM7 [35,36]. Among them, NS8593 is suggested as a potent TRPM7 inhibitor [20]. Structural analyses revealed that NS8593 binds to a vanilloid-like site, stabilizing the closed conformation and thereby suppressing channel activity [37]. Moreover, NS8593 treatment reduces TRPM7 autophosphorylation and attenuates kinase-dependent signaling pathways [38,39]. Our results indicate that pharmacological inhibition of TRPM7 via NS8593 recapitulates the key phenotypes observed in *Trpm7*^*R/R*^ mice. Specifically, NS8593 treatment impairs rpS6 phosphorylation in pancreatic islets isolated from WT mice. This observation is consistent with previous studies reporting that NS8593 suppresses the mTOR signaling pathway in various cell types [10,29]. Furthermore, NS8593 exposure elicited a marked disruption in α-cell transcriptional programming, significantly downregulating the expression of *Gcg* and key transcription factors including *Mafb*, *FoxA2*, *Nkx2.2*, and *Pou3f4*, which are essential for α-cell development and maintenance. This transcriptional dysregulation was accompanied by a decrease in α-cell proliferation and an increase in apoptosis, culminating in reduced α-cell mass. Although NS8593-mediated TRPM7 inhibition may influence cytosolic Mg^2+^ levels, the phenotypic similarity between NS8593-treated and *Trpm7*^*R/R*^ islets indicates that TRPM7 kinase and its downstream signaling are the principal drivers of α-cell regulation.

Emerging evidence suggests that the mTORC1–FoxA2 axis is a crucial link between nutrient sensing and transcriptional regulation in α-cells. FoxA2, a transcription factor essential for preserving α-cell phenotypic identity and function, appears to act downstream of mTORC1, regulating the expression of genes involved in glucagon synthesis such as *Gcg* and glucagon secretion [2,40]. These findings collectively point to TRPM7 as a potential upstream regulator of the mTORC1–FoxA2 axis.

As a final step, we used the αTC1c9 cell line to exclude potential confounding influences from paracrine signaling or cell–cell interactions present in intact islets. Patch-clamp recordings confirmed functional TRPM7 channel activity in αTC1c9 cells, which NS8593 effectively inhibited. Treatment of these cells with NS8593 recapitulated the *in vivo* and *ex vivo* phenotypes, including impaired α-cell identity gene expression, reduced proliferation, and increased apoptosis. This cell-autonomous effect firmly establishes TRPM7 kinase as a direct regulator of α-cell biology, independent of external microenvironmental influences.

In conclusion, our findings uncovered a previously unknown mechanistic link between TRPM7 kinase activity and the mTORC1/FoxA2 signaling axis. Furthermore, we clarified how TRPM7 regulates the expression of genes essential for glucagon biosynthesis and α-cell function. We demonstrate that TRPM7 kinase plays an essential role in regulating α-cell mass and glucagon secretion, potentially through modulation of the mTOR signaling pathway. Collectively, these results establish TRPM7 as a central regulator of α-cell physiology and suggest a key role in preserving α-cell phenotypic identity and functional integrity. Despite these mechanistic insights into TRPM7 kinase function in α-cell biology, several limitations warrant consideration. Notably, the genetic model used involves systemic inactivation of TRPM7 kinase activity rather than α-cell-specific targeting. Therefore, indirect effects arising from TRPM7 dysfunction in non-α-cell populations cannot be fully excluded. Nevertheless, the close alignment between the phenotypes observed in *Trpm7*^*R/R*^ islets and those induced by acute pharmacological inhibition of TRPM7 in an established α-cell model supports a predominantly cell-autonomous role for TRPM7 in regulating mTOR signaling and α-cell function. Future studies employing α-cell-specific deletion of TRPM7 will be required to define the tissue-specific contributions of this chanzyme to α-cell physiology.

## CRediT authorship contribution statement

**Severin Boulassel:** Writing – review & editing, Visualization, Methodology, Investigation, Formal analysis. **Pascale C.F. Schreier:** Writing – review & editing, Methodology, Investigation. **Andreas Beck:** Writing – review & editing, Methodology, Investigation. **Hyeri Choi:** Writing – review & editing, Methodology, Investigation. **Anna M. Melyshi:** Methodology, Investigation. **Peter S. Reinach:** Writing – review & editing. **Megan Duraj:** Methodology. **Mikhail Vinogradov:** Methodology, Formal analysis. **Bibiazhar Suleimen:** Methodology, Investigation, Formal analysis. **Johanna Berger:** Methodology, Investigation. **Katharina Jacob:** Methodology. **Andreas Breit:** Writing – review & editing. **Susanna Zierler:** Writing – review & editing. **Ingrid Boekhoff:** Writing – review & editing. **Thomas Gudermann:** Writing – review & editing, Resources, Funding acquisition, Conceptualization. **Noushafarin Khajavi:** Writing – original draft, Visualization, Validation, Supervision, Software, Resources, Project administration, Methodology, Investigation, Funding acquisition, Formal analysis, Data curation, Conceptualization.

## Declaration of competing interest

The authors declare no competing interest.

## Data Availability

Data will be made available on request.
