# Supplementary material for: TRPM7 kinase regulates α-cell proliferation and glucagon production in mice
Source: Mol Metab. 2026 Jan 9;104:102317. doi: 10.1016/j.molmet.2026.102317 (PMC12860271; doi:10.1016/j.molmet.2026.102317)
Supplement: Multimedia component 1 [file mmc1.docx]

| Antigen | Host species | Dilution | Source | Catalogue number | Application |
| --- | --- | --- | --- | --- | --- |
| Glucagon | Mouse | 1:2000 | Sigma Aldrich | G2654 | IF |
| Ki67 | Rabbit | 1:200 | Abcam | Ab15580 | IF |
| Alexa Fluor 488 goat anti-mouse | Goat | 1:1000 | Thermo Fisher Scientific | A11001 | IF |
| Alexa Fluor 633 goat anti-mouse | Goat | 1:500 | Thermo Fisher Scientific | A21052 | IF |
| Alexa Fluor 647 goat anti-rabbit | Goat | 1:1000 | Thermo Fisher Scientific | A21245 | IF |
| Histone H3 | Rabbit | 1:10000 | Abcam | Ab1791 | WB |
| pS6 | Rabbit | 1:1000 | Cell Signaling | 5364 | WB |
| S6 | Rabbit | 1:1000 | Cell Signaling | 2217 | WB |
| Goat Anti-Rabbit IgG (H + L) HRP Conjugate | Goat | 1:5000 | Bio-Rad | 1706515 | WB |

**Suppl. Table 1. Antibody descriptions**

| Gene | Primer sequence | Amplicon (bp) |
| --- | --- | --- |
| *Gcg* | Forward: GTCTACACCTGTTCGCAGCT  Reverse: CTCTGTGTCTTGAAGGGCGT | 125 |
| *Mafb* | Forward: GTGTTCTGCCTTCCTCCTCC  Reverse: GTGGGCTACTCCTCTCAGGA | 137 |
| *FoxA2* | Forward: CAGCTACTACGCGGAGCC  Reverse: GCTCATTCCAGCGCCCAC | 200 |
| *Nkx2.2* | Forward: TCGCTCTCCCCTTTGAACTTT  Reverse: GTTAACGTTGGGATGGTTTGG | 121 |
| *Pou3f4* | Forward: GGTGCGTGTCTGGTTCTGTA  Reverse: CACCTCCTTGCTTCCTCCAG | 144 |
| *Pax6* | Forward: CTGAGGAACCAGAGAAGACAGG  Reverse: CATGGAACCTGATGTGAAGGAGG | 132 |
| *Pcsk2* | Forward: ACCTCTTTGGCTACGGAGTCCT  Reverse: TTGAGGGTCAGTACCAGCTTGC | 163 |

**Suppl. Table 2. qRT-PCR primers**

**Suppl. Table 3. Relevant downregulated genes in *Trpm7^R/R^* mice**

| Gene | Fold change | P value |
| --- | --- | --- |
| Gcg | -3.793622771 | 0.000003050 |
| Mafb | -2.2494562 | 0.010578146 |
| Ttr | -5,422245735 | 0.0000001911 |
| Pcsk2 | -2.781563877 | 0.000372079 |
| Pax6 | -2.043828505 | 0.011728595 |
| Abcc8 | -2.545207419 | 0.002565963 |
| Hsp90ab1 | -4.007202505 | 0.00001083 |


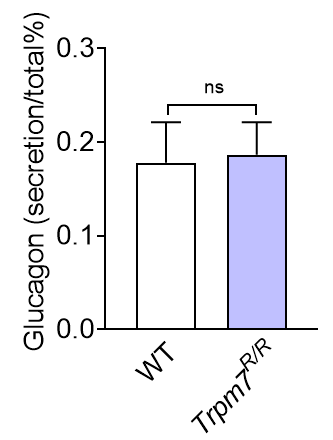


**Supplemental Figure. 1 Glucagon secretory responses to KCl treatment in WT and *Trpm7^R/R^* islets.** Percentage of glucagon content secreted from intact WT or *Trpm7^R/R^* islets after incubation with KCl (30 mM). Data are given as means ± SEM, and statistical difference was assessed by unpaired 2-tailed Student’s t test.

**
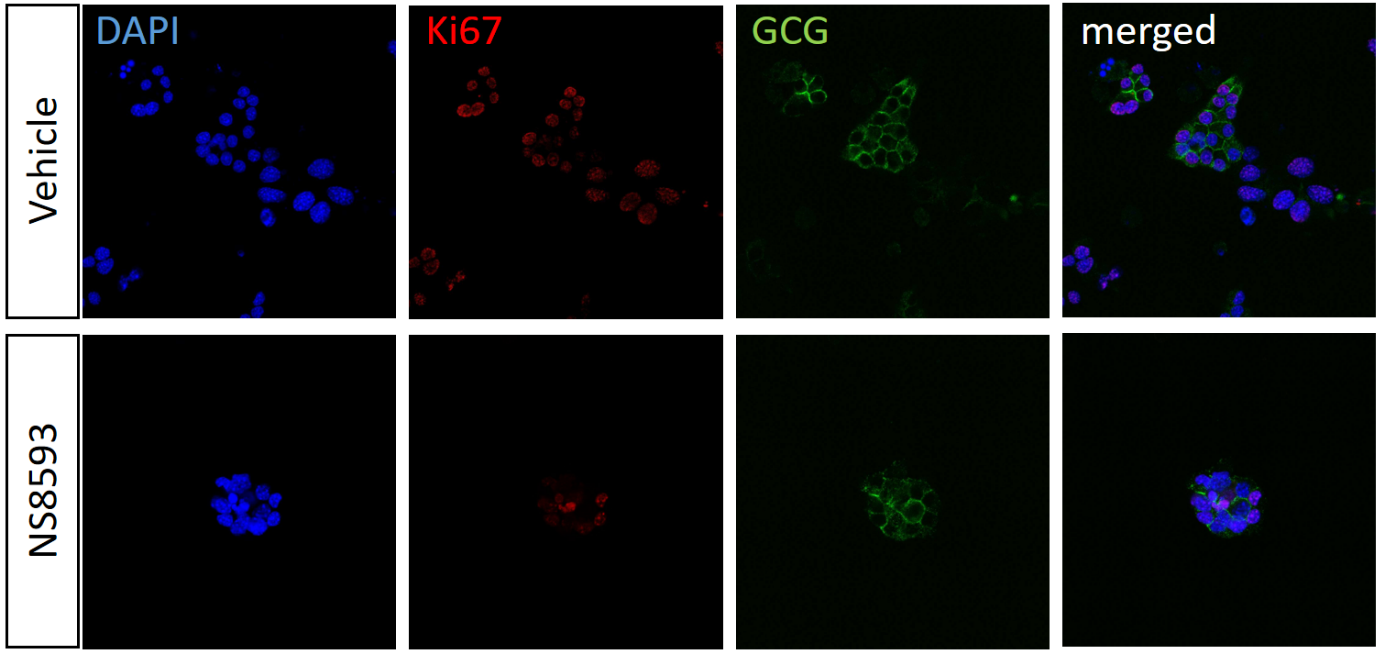
Supplemental Figure. 2** Confocal images of αTC1c9 cells stained for DAPI (blue), glucagon (green), and Ki67 (red). The αTC1c9 cells were treated with DMSO (Vehicle) or 30 µM NS8593 for 48 h.


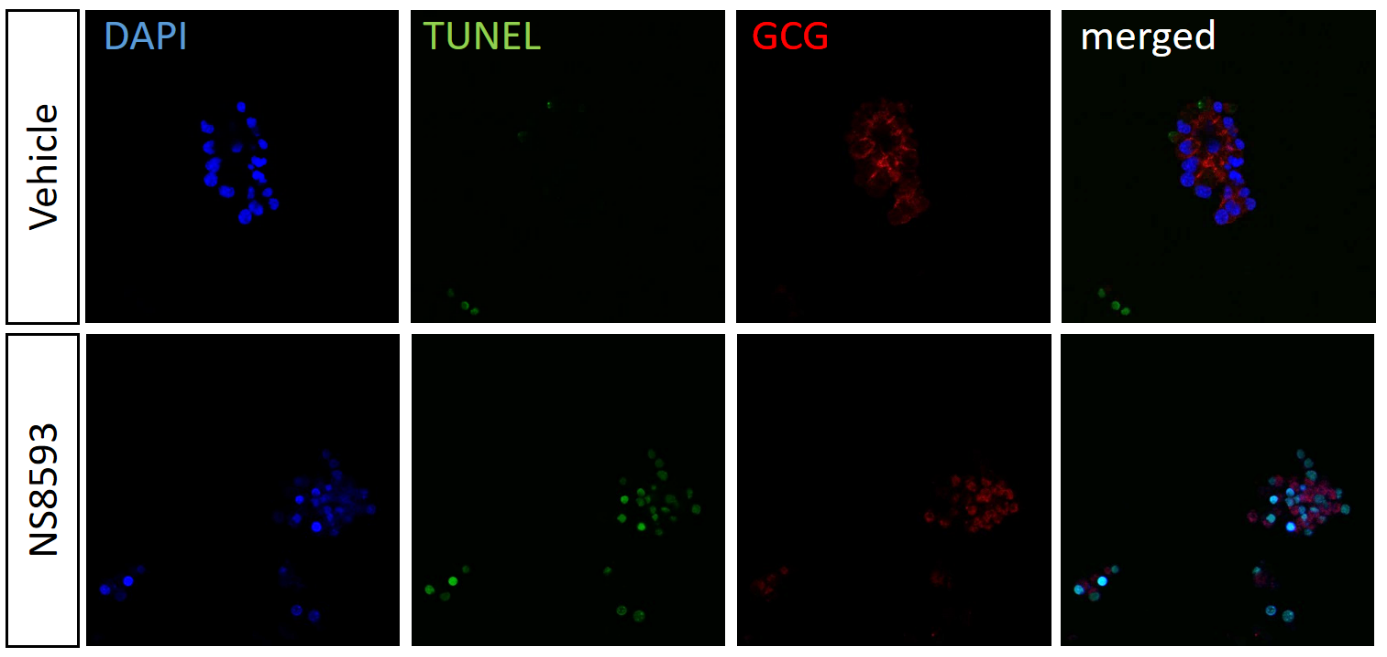


**Supplemental Figure. 3** Confocal images of TUNEL staining of αTC1c9 cells. TUNEL staining is shown in green, glucagon staining is shown in red, and nuclei (DAPI) are shown in blue. The αTC1c9 cells were treated with DMSO (Vehicle) or 30 µM NS8593 for 48 h.
